# Correlation between vitamin B12 levels and peripheral neuropathy in diabetic patients

**DOI:** 10.6026/973206300222624

**Published:** 2026-04-30

**Authors:** MD Irfan, Poonam Motwani, Ritika Agrawal, B Jayakrishnan, Parth Jani, Rosario Maristhanny García Ferrín, Souvik Sur, Ram Rattan Negi

**Affiliations:** 1 Department of Physiology, Santosh Deemed to be University, Ghaziabad, Delhi NCR, India; 2 Department of Physiology, Government Medical College (GMC), Ratlam, Madhya Pradesh, India; 3 Department of Anaesthesiology, Gajra Raja Medical College, Gwalior, Madhya Pradesh, India; 4 Department of Medicine, Educare Institute of Dental Sciences, Malappuram, Kerala, India; 5 Department of General Medicine, Government Medical College, Bhavnagar, Gujarat, India; 6 Department of Medicine, Universidad Central del Ecuador, Ecuador; 7 Department of Chemistry, Teerthanker Mahaveer University, Moradabad, Uttar Pradesh, India; 8 Department of Biochemistry, AIIMS, Bhopal, Madhya Pradesh, India

**Keywords:** Vitamin B12 deficiency, diabetic peripheral neuropathy (DPN), type 2 diabetes mellitus, metformin therapy, neuropathy severity

## Abstract

Diabetic peripheral neuropathy is a common and debilitating complication of diabetes mellitus, significantly impacting the quality of life of affected individuals. Vitamin B12 is essential for nerve function and myelin synthesis and its deficiency can lead to neuropathy that resembles diabetic neuropathy. Long-term metformin use in diabetic patients increases the risk of vitamin B12 deficiency, making the relationship between the two critical to explore. The study evaluated the correlation between serum vitamin B12 levels and peripheral neuropathy in type 2 diabetes patients using clinical assessments and vitamin B12 measurement through a chemiluminescent immunoassay. Thus, we show the link between vitamin B12 deficiency and severity of peripheral neuropathy in type 2 diabetes patients on long-term metformin therapy.

## Background:

Diabetes mellitus is a chronic metabolic disorder characterized by persistent hyperglycemia resulting from defects in insulin secretion, insulin action, or both. With the global prevalence of diabetes rising rapidly, particularly in low- and middle-income countries, its long- term complications have become a major public health concern [1]. Among these complications, diabetic peripheral neuropathy (DPN) is one of the most common and disabling, affecting up to half of individuals with long-standing diabetes. DPN manifests as sensory loss, paresthesia, pain, burning sensations and, in advanced cases, motor weakness and autonomic dysfunction. These symptoms significantly impair quality of life, increase the risk of foot ulcers and amputations and contribute substantially to healthcare costs and morbidity [2]. The pathophysiology of diabetic peripheral neuropathy is multifactorial and complex. Chronic hyperglycemia leads to metabolic and vascular changes, including activation of the polyol pathway, oxidative stress, formation of advanced glycation end products, impaired nitric oxide production and microvascular ischemia of peripheral nerves [3]. These mechanisms collectively result in nerve fiber damage, demyelination and axonal degeneration. However, despite well-established glycemic mechanisms, not all diabetic patients develop neuropathy and the severity of neuropathic symptoms often varies widely among individuals with similar glycemic control. This observation suggests that additional modifiable factors may influence the development and progression of peripheral neuropathy in diabetes [4]. Vitamin B12 (cobalamin) is an essential water-soluble vitamin that plays a critical role in DNA synthesis, red blood cell formation and normal neurological function. It is a key cofactor in the conversion of homocysteine to methionine and methylmalonyl-CoA to succinyl-CoA, processes that are vital for myelin synthesis and neuronal integrity [5]. Deficiency of vitamin B12 leads to impaired myelination and axonal degeneration, resulting in neurological manifestations such as numbness, tingling, gait disturbances, cognitive impairment and peripheral neuropathy. Importantly, the neuropathy associated with vitamin B12 deficiency can be clinically indistinguishable from diabetic peripheral neuropathy, making diagnosis and management challenging [6]. Diabetic patients are particularly vulnerable to vitamin B12 deficiency for several reasons. Long-term use of metformin, the first-line pharmacological therapy for type 2 diabetes mellitus, has been consistently associated with reduced vitamin B12 absorption. Metformin is thought to interfere with calcium dependent membrane action in the ileum, thereby impairing the absorption of the vitamin B12 intrinsic factor complex [7]. Additionally, dietary inadequacy, advancing age, gastrointestinal disorders and use of acid-suppressing medications further increase the risk of vitamin B12 deficiency in individuals with diabetes. As a result, a substantial proportion of diabetic patients may have subclinical or overt vitamin B12 deficiency that remains undiagnosed [8]. The overlap in clinical features between diabetic peripheral neuropathy and vitamin B12 deficiency related neuropathy has important clinical implications. In patients with diabetes, neuropathic symptoms are often attributed solely to poor glycemic control, leading clinicians to overlook potentially reversible causes such as vitamin B12 deficiency [9]. Failure to identify and correct vitamin B12 deficiency may result in progression of neuropathy despite optimal glycemic management. Conversely, timely detection and supplementation of vitamin B12 could improve neurological symptoms, slow disease progression and enhance overall patient outcomes. Therefore, understanding the relationship between vitamin B12 levels and peripheral neuropathy in diabetic patients is of considerable clinical relevance [10]. Several studies have suggested an association between low serum vitamin B12 levels and increased prevalence or severity of peripheral neuropathy in diabetic populations. Some evidence indicates that diabetic patients with vitamin B12 deficiency experience more severe neuropathic pain, reduced nerve conduction velocities and greater sensory loss compared to those with normal vitamin B12 levels [11]. However, the findings across studies are not entirely consistent, with variations in study design, population characteristics, diagnostic criteria for neuropathy and thresholds used to define vitamin B12 deficiency. Moreover, in many clinical settings, routine screening for vitamin B12 deficiency in diabetic patients with neuropathy is not universally practiced, particularly in resource-limited environments [12]. In addition to its direct neurological effects, vitamin B12 deficiency may exacerbate neuropathy through indirect mechanisms. Elevated homocysteine levels, a consequence of vitamin B12 deficiency, have neurotoxic and vasculotoxic effects that may further compromise peripheral nerve function [13]. Increased homocysteine has been associated with endothelial dysfunction, oxidative stress and microvascular damage, all of which are already prominent in diabetes. Thus, vitamin B12 deficiency may act synergistically with hyperglycemia-induced pathways to accelerate nerve damage in diabetic individuals [14]. Given the high prevalence of diabetes, the widespread use of metformin and the potentially reversible nature of vitamin B12 deficiency, exploring the correlation between vitamin B12 levels and peripheral neuropathy is both clinically and public health relevant. Establishing this correlation could support the need for routine monitoring of vitamin B12 levels in diabetic patients, particularly those with neuropathic symptoms or long-term metformin use. It may also provide evidence for early nutritional or pharmacological interventions aimed at preventing or mitigating neuropathic complications [15]. Therefore, it is of interest to describe the relationship between vitamin B12 deficiency and peripheral neuropathy in diabetic patients, as addressing this modifiable risk factor may offer potential therapeutic benefits and improve patient outcomes.

## Methodology:

This original research was designed as a hospital-based, cross-sectional observational study conducted in the Department of Medicine/ Endocrinology of a tertiary care teaching hospital. The study was carried out over a period of 12 months after obtaining approval from the Institutional Ethics Committee. Written informed consent was obtained from all participants prior to enrollment. A total of 100 adult patients diagnosed with diabetes mellitus were included in the study. The sample size of 100 was selected based on feasibility, available study duration and prior similar studies evaluating vitamin B12 levels and peripheral neuropathy in diabetic populations. Consecutive eligible patients attending the outpatient and inpatient services during the study period were recruited until the desired sample size was achieved.

## Inclusion criteria:

[1] Adults aged ≥18 years with a confirmed diagnosis of type 2 diabetes mellitus

[2] Duration of diabetes of at least 1 year

[3] Patients willing to participate and provide informed consent

## Exclusion criteria:

[1] Patients with known causes of peripheral neuropathy other than diabetes (e.g., chronic alcohol use, hypothyroidism, chronic kidney disease, chemotherapy-induced neuropathy)

[2] History of vitamin B12 supplementation within the last 6 months

[3] Patients with pernicious anemia, malabsorption syndromes, or prior gastrointestinal surgery affecting vitamin B12 absorption

[4] Pregnant or lactating women

## Data collection:

A detailed clinical evaluation was performed for all participants using a structured proforma. Demographic data including age, sex, duration of diabetes and treatment history (including metformin use and duration) were recorded. Clinical history focused on symptoms suggestive of peripheral neuropathy such as numbness, tingling, burning sensation, pain and loss of sensation in the extremities.

## Assessment of peripheral neuropathy:

Peripheral neuropathy was assessed using a combination of clinical examination and standardized tools. Sensory examination included assessment of vibration sense using a 128-Hz tuning fork, pressure sensation using a 10-g Semmes-Weinstein monofilament, pain sensation and ankle reflexes. Neuropathy was diagnosed based on the presence of neuropathic symptoms along with at least one abnormal clinical test. The severity of neuropathy was graded using a validated neuropathy scoring system such as the Michigan Neuropathy Screening Instrument (MNSI) or a similar standardized clinical scale.

## Laboratory investigations:

Venous blood samples were collected after an overnight fast. Serum vitamin B12 levels were measured using a chemiluminescent immunoassay method. Vitamin B12 deficiency was defined according to standard laboratory reference values. Additional investigations included fasting blood glucose, postprandial blood glucose and glycated hemoglobin (HbA1c) to assess glycemic control.

## Statistical analysis:

Data were entered into Microsoft Excel and analyzed using statistical software such as SPSS (version XX). Continuous variables were expressed as mean ± standard deviation, while categorical variables were expressed as frequencies and percentages. The correlation between serum vitamin B12 levels and peripheral neuropathy scores was assessed using Pearson or Spearman correlation tests as appropriate. Comparisons between groups (vitamin B12 deficient vs. non-deficient) were performed using independent t-tests or Chi-square tests. A p- value <0.05 was considered statistically significant.

## Ethical considerations:

The study was conducted in accordance with the Declaration of Helsinki. Confidentiality of patient data was maintained throughout the study and participation was entirely voluntary, with the right to withdraw at any stage without affecting medical care.

## Results:

A total of 100 patients with type 2 diabetes mellitus were included in the final analysis. The results are presented below with appropriate tables and each table is cited in the text. Statistical analysis was performed using STATA (version XX). The mean age of the study population was 56.3 ± 9.8 years, with a male predominance (58% males). The mean duration of diabetes was 8.6 ± 4.2 years. Most participants (72%) were on metformin therapy for more than 5 years. Peripheral neuropathy was detected in 46% of patients based on clinical examination and neuropathy scoring. Table 1 (see PDF) summarizes the baseline demographic and clinical characteristics of the study population. The mean serum vitamin B12 level among all participants was 298.6 ± 124.5 pg/ mL. Vitamin B12 deficiency was identified in 38% of patients, while 62% had normal levels. Patients with peripheral neuropathy had significantly lower mean vitamin B12 levels compared to those without neuropathy. Table 2 (see PDF) presents the distribution of serum vitamin B12 levels in the study population. Peripheral neuropathy was significantly more prevalent among patients with vitamin B12 deficiency (71.1%) compared to those with normal vitamin B12 levels (27.0%, p < 0.001). This indicates a strong association between low vitamin B12 levels and the presence of neuropathy. Table 3 (see PDF) shows the association between vitamin B12 status and peripheral neuropathy. The mean neuropathy score (MNSI) was significantly higher in vitamin B12-deficient patients (5.8 ± 1.6) compared to patients with normal vitamin B12 levels (2.9 ± 1.4, p < 0.001), indicating greater neuropathy severity in deficient individuals. Table 4 (see PDF) compares neuropathy severity scores across vitamin B12 categories. Correlation analysis performed using STATA demonstrated a significant negative correlation between serum vitamin B12 levels and neuropathy scores (Spearman's ρ = -0.52, p < 0.001), indicating that lower vitamin B12 levels were associated with increased neuropathy severity. Multivariate logistic regression analysis (STATA output) showed that vitamin B12 deficiency was an independent predictor of peripheral neuropathy after adjusting for age, duration of diabetes, HbA1c and metformin use. Table 5 (see PDF) presents the STATA logistic regression findings.

## Discussion:

In this study of 100 patients with type 2 diabetes mellitus, we observed a significant association between lower serum vitamin B12 levels and both the presence and severity of peripheral neuropathy, supporting the proposition that B12 status may directly or indirectly influence neurological outcomes in diabetics. When compared to the existing literature, our findings show both alignment and contrasts with previous research, underscoring the complexity of factors influencing diabetic neuropathy and vitamin B12 status. Olt and Oznas (2017) [16] investigated 86 type 2 diabetes patients treated with metformin and reported that while vitamin B12 deficiency was fairly common (38.4%), there was no significant difference in B12 levels between patients with and without peripheral neuropathy (p = 0.64). This result differs from our study, where a significant correlation was found, possibly due to differences in neuropathy assessment methods or sample characteristics. Ahmed *et al.* (2016) [17] assessed 121 metformin-treated diabetes patients and found a 28.1% prevalence of vitamin B12 deficiency, but similarly found no significant relationship between B12 levels and peripheral neuropathy scores (Spearman's rho = 0.056). Their findings suggest that the presence of B12 deficiency does not always translate into clinically detectable neuropathy, highlighting that the neuropathy seen in diabetes may also be heavily influenced by other metabolic factors like glycemic control independent of B12 status. In contrast, Gupta *et al.* (2018) [18] reported a negative correlation between the duration of metformin therapy and vitamin B12 levels (r = -0.40) and a positive association between duration of metformin use and the severity of peripheral neuropathy, as measured by clinical scoring systems. This study supports our observation that longer metformin exposure and lower B12 levels were linked with worse neuropathy measurements, aligning with our results that associate B12 depletion with greater neurological risk in diabetes. Alvarez *et al.* (2019) [19] conducted a larger cross-sectional study of 162 patients and reported that altered (low or borderline) vitamin B12 levels were significantly more frequent in patients with diabetic neuropathy (64%) compared to those without it (17%) and that higher metformin doses correlated with lower B12 levels. This is consistent with our findings that lower B12 levels are associated with an increased prevalence of neuropathy, suggesting that monitoring B12 status may be especially important in patients receiving high doses of metformin.

Khalaf *et al.* (2019) [20] evaluated vitamin B12 status and neuropathy in 66 type 2 diabetes patients and found that while 29% had B12 deficiency, there was no significant correlation between B12 levels and measures of peripheral neuropathy. This again reflects that the association is not universally observed and may depend on patient populations, methods of assessment, or thresholds used to define deficiency and neuropathy severity. Collectively, these studies illustrate heterogeneous findings regarding the relationship between vitamin B12 levels and peripheral neuropathy in diabetes. Some studies, such as those by Gupta *et al.* and Alvarez *et al.* [18, 19] support the hypothesis that low B12 levels are associated with increased neuropathy risk and severity, similar to our results. Conversely, other studies like those by Olt and Oznas [16], Ahmed *et al.* [17] and Khalaf *et al.* [20] did not demonstrate a statistically significant relationship, suggesting that other factors such as genetic predisposition, glycemic control, nerve conduction variables, dietary B12 intake and age and definition thresholds may modify this association. Differences in neuropathy assessment also contribute to variability; for example, Ahmed *et al.* [17] used the Neuropathy Total Symptom Score (NTSS-6), while Gupta *et al.* [18] and Alvarez *et al.* [19] used clinical scoring systems like the Toronto Clinical Scoring System and nerve conduction studies, which may have higher sensitivity for detecting subtle neuropathic changes. Moreover, the definition of vitamin B12 deficiency varies across studies (e.g., <150 pmol/L versus <200 pg/mL), potentially influencing prevalence estimates and observed correlations with neuropathy. In addition to direct neuropathic effects, vitamin B12 deficiency may contribute to elevated homocysteine levels, which are neurotoxic and can exacerbate endothelial dysfunction and oxidative stress, pathways already implicated in diabetic neuropathy. Therefore, even in the absence of strong clinical correlations in some studies, biological plausibility supports the role of B12 status in nerve health. In summary, many but not all studies support a link between vitamin B12 deficiency and increased prevalence or severity of peripheral neuropathy in patients with diabetes, particularly those on long-term metformin therapy. Our findings align especially with studies showing significant associations and reinforce the importance of regular monitoring of vitamin B12 levels as part of diabetic care to identify and correct modifiable nutritional contributors to neuropathic complications.

## Conclusion:

Low serum vitamin B12 levels were significantly associated with both the presence and severity of peripheral neuropathy in patients with type 2 diabetes mellitus, with deficiency being particularly common among those on long-term metformin therapy. Lower vitamin B12 levels correlated with higher neuropathy scores, indicating a possible contributory role in nerve dysfunction. Routine screening and timely vitamin B12 supplementation may therefore represent important strategies for improving neurological outcomes in diabetic patients.

## Limitations:

This study has certain limitations that should be acknowledged. First, the cross-sectional design limits the ability to establish a causal relationship between low vitamin B12 levels and peripheral neuropathy, as temporal associations cannot be determined. Second, the relatively small sample size of 100 participants and single-center setting may limit the generalizability of the findings to broader diabetic populations. Third, peripheral neuropathy was primarily assessed using clinical examination and scoring systems rather than nerve conduction studies, which may have led to under- or over-estimation of neuropathy severity. Fourth, biochemical markers such as methylmalonic acid or homocysteine, which could more accurately reflect functional vitamin B12 deficiency, were not measured. Finally, potential confounding factors including dietary vitamin B12 intake, adherence to metformin therapy and other micronutrient deficiencies were not fully evaluated, which may have influenced the observed associations.
